# Duration of a cow-milk exclusion diet worsens parents’ perception of quality of life in children with food allergies

**DOI:** 10.1186/1471-2431-13-203

**Published:** 2013-12-05

**Authors:** Luciana Indinnimeo, Luciano Baldini, Valentina De Vittori, Anna Maria Zicari, Giovanna De Castro, Giancarlo Tancredi, Giulia Lais, Marzia Duse

**Affiliations:** 1 Department of Pediatrics, Service of Pediatric Immunology and Allergy, “Sapienza”, University of Rome, Rome, Italy; 2 Department of Psychology of the Processes of Development and Socialization, “Sapienza” University of Rome, Rome, Italy

**Keywords:** Quality of life, Food allergy, Questionnaire, Diet, Milk, Egg, Food-related anxiety, Italian

## Abstract

**Background:**

In Italy, rigorous studies obtained with specific and validated questionnaires that explore the impact of exclusion diets on health-related quality of life (HRQoL) in children with food allergies are lacking. In this cross-sectional study, we wished to validate the Italian version of a disease-specific quality of life questionnaire, and assess the impact of exclusion diets on the HRQoL in a cohort of Italian children with IgE-mediated food allergies.

**Methods:**

Children on an exclusion diet for ≥1 food were enrolled consecutively, and their parents completed the validated Italian version of the Food Allergy Quality of Life Questionnaire–Parent Form (FAQLQ-PF) and Food Allergy Independent Measure (FAIM).

**Results:**

Ninety-six parents of children aged 0–12 years answered the FAQLQ–PF. The validity of the construct of the questionnaire was assessed by correlation between the FAQLQ–PF and FAIM–PF (r = 0.85). The Italian version of the FAQLQ had good internal consistency (Cronbach's α >0.70). Factors that mainly influenced the HRQoL were older age, severity of food allergy, and the duration of the cow milk-exclusion diet.

**Conclusions:**

The FAQLQ–PF, validated in Italian, is a reliable instrument. Worse QoL scores were observed among older children, those with severe systemic reactions, and those with a prolonged cow milk-free diet. It is very important to consider the QoL assessment as an integral part of food-allergy management. These results emphasize the need to administer exclusion diets only for the necessary time and the importance of assessment of the HRQoL in these patients.

## Background

Food allergy is an adverse immune response to food proteins. It is sustained by three immunological mechanisms: immunoglobulin (Ig)E-mediated, cell-mediated, and mixed (IgE- and cell-mediated) [1]. A meta-analysis of patients allergic to milk, eggs, nuts and fish supported by oral provocation tests showed a global prevalence of ≈2–3.5 [2].

In the USA, Branum and Lukacs [3] reported an 18% increase in the prevalence of food allergies in children and under-18s from 1997 to 2007, and a threefold increase in outpatient visits in 2006 compared with 1993. In 2007, ≈4% of children were reported to suffer from food-related allergies. With regard to Italy, Caffarelli et al. [4], in a study of in 625 children aged 5–14 years, discovered a prevalence and incidence of adverse reactions to food over a lifetime of 10.5% and 1.6%, respectively.

The specificity and course of immune responses determine the physical manifestations. These include acute life-threatening events such as anaphylaxis, or chronic and debilitating manifestations such as atopic dermatitis and eosinophilic gastroenteropathy. Treatment for food allergy is based on educating patients about strict exclusion diets to prevent ingestion of the allergen and on emergency treatment plans for accidental reactions. Novel approaches include sublingual/oral immunotherapy, monoclonal anti-IgE antibodies, cytokines/anti-cytokines, as well as Chinese herbal therapies [5].

Health-related quality of life (HRQoL) is a multidimensional construct comprising physical, psychological and social components. It reflects the experience of illness and therapies as perceived by the child and his/her family in their everyday lives; it may be a predictor of therapeutic success and may have strong prognostic importance. Chronically ill children and their families experience stress that leaves them at risk of developing long-term physical, emotional, and psychosocial problems; this may negatively influence their HRQoL [6]. Problems are often more pronounced during the school years and adolescence because psychosocial development is influenced by social engagement with families and peers, and it is important not to be “too different” from other children [7,8]. Olsson et al. found that negotiating relationships with peers is a central aspect of managing chronic illness in adolescence, as are young people’s emotional responses to chronic illness, their acceptance of illness, and their efforts to find “meaning” out of having a chronic illness [9].

The influence of asthma and rhinitis on daily life has been investigated thoroughly [10-12]. However, there are few data on the impact of treatment for food allergy on the HRQoL of children and their families because few studies have investigated the effect of food allergy [13-15]. Avery et al. [13] compared the HRQoL in subjects with diabetes mellitus (DM) and patients with allergies to peanuts. The results showed that patients allergic to peanuts experienced a poorer HRQoL than the other group even though DM patients had a chronic debilitating disease. This result is not entirely surprising given the high risk of severe (potentially fatal) anaphylaxis in the peanut-allergy group. Marklund et al. [14] studied the parent-reported HRQoL in the families of 134 schoolchildren considered to have food hypersensitivity (allergy or intolerance). Food hypersensitivity by itself was associated with deterioration of the child's psychosocial HRQoL, regardless of additional allergic disease, suggesting that it is the risk of food reactions and measures to avoid them that are associated with lower HRQL, rather than the clinical reactivity induced by food intake.

The Food Allergy Quality of Life Questionnaire–Parent Form (FAQLQ–PF) is a food allergy-specific questionnaire that has recently been developed and validated in English [16,17]. The aim of the present study was to validate the Italian version of this disease-specific questionnaire. Then, we assessed the impact of exclusion diets on parent-perceived HRQoL in an Italian pediatric population of different ages with IgE-mediated food allergy.

## Methods

This was a cross-sectional study in which children with food allergy were recruited consecutively during clinical visits. Parents were provided with verbal information by a physician. Parents then provided written informed consent. Approval was obtained from the Department of Pediatrics as well as the Pediatric Neuropsychiatry Department of Policliclino Umberto 1 of Rome. The parents of recruited children who agreed to participate in the study were provided with the Italian version of the FAQLQ–PF. The questionnaire was completed by the parents at the end of the clinic visit. A psychologist was able to provide explanations if required.

We recruited children aged <12 years who had documented IgE-mediated food allergy, were on an elimination diet, and who had an outpatient appointment at the Pediatric Service of Immunology and Allergology at Umberto 1 Hospital (Rome, Italy) between April 2010 and July 2011. Food allergy was diagnosed previously by a consultant in pediatric allergy and confirmed with an open challenge for ≥1 nutrients with a positive skin test.

In children with recent episodes of anaphylaxis (an oral challenge is not indicated in such patients), food allergy was diagnosed by a convincing history of severe allergic reactions supported by evidence of sensitization to a specific food according to current guidelines [18,19].

After obtaining parental consent, the challenge was conducted by administering standardized increasing doses of food. The test was considered to be positive if at least one of these manifestations appeared: urticaria, laryngospasm, wheezing, abdominal pain, diarrhea or vomiting. Patients with severe chronic conditions such as uncontrolled asthma (as defined by asthma guidelines [20]) or atopic dermatitis requiring regular use of oral corticosteroids were excluded to prevent the impact of the other chronic diseases on the HRQoL.

### FAQLQ–PF Questionnaire

The FAQLQ–PF Questionnaire was translated from English into Italian by a team of psychologists whose native language was Italian. To ensure accurate translation from the original English questionnaire, the English and Italian translations were given to three bilingual medical experts. We observed a significant positive correlation (r > 0.9; p < 0.001) between the answers to each item. Then, a third bilingual medical expert whose native language was English back-translated the questionnaire from Italian to English.

The questionnaire [16] has six sections (A–F). To assess the HRQoL of children, we used sections A–C. The other sections investigate anamnestic information about the: clinical history of children and their families; type of food allergy; symptoms that occurred after intake of the specific food. The last section of the questionnaire investigates the concerns of parents about their child’s illness. Sections A–C contain items which refer to three sub-scores: evaluation of emotional impact; food anxiety; social and dietary limitations. For each item, parents completing the form can choose one of six answers on a six-step Likert scale.

This questionnaire is divided into three age groups. It addresses a different number of questions according to the child’s age. That is, parents of children aged 0–3 years respond only to section A (14 items); parents of 4–6-year-old children respond to sections A and B (26 items); parents of 7–12-year-old children respond to sections A–C (30 items). The higher the score of the items in sections A–C, the greater the extent that emotional impact, food-related anxiety and social limitations interfere with daily activities, and have a negative impact on the HRQoL. The FAQLQ was chosen in the parent form to include children of very early age, when food allergies are particularly common. There was no control group with healthy children because this is not applicable if using a disease-specific questionnaire.

### Food Allergy Independent Measure (FAIM) questionnaire

The FAIM questionnaire was validated by van der Velde et al. [21]. It is a measurement of the severity of food allergy and was developed to evaluate the construct validity of FAQLQs. The questionnaire contains six questions scored on a seven-point scale (0 to 6). Questions 2 and 3 were adapted from questions which were developed for validation of the Vespid Allergy Quality of Life Questionnaire (VQLQ) [22] and Food Allergy Quality of Life Parental Burden Questionnaire (FAQL–PB) [23]. Questions 1 and 4 were likely to be an additional source of HRQoL differences in patients with food allergies, and were developed. Additionally, two independent-measure questions (IM 1 and 2) were developed. These questions reflect aspects of the perceived severity of food allergy not captured by the other four questions. The translation procedure, as described for the FAQLQ–PF, was followed for the FAIM–PF as well.

### Statistical analyses

To ensure the validity of the translated questionnaire, 45 participants were taken into consideration. The sample size (m) of the study was calculated: m = (2c/δ2) +1 where δ = │μ2 – μ1│/σ μ1 and μ2 represent the means of treatment and control group, respectively, σ is the standard deviation, and δ is the effect size (assuming Total QoL μ = 2.0).

Hence, if we assume a power of 80% and a level of significance set at 5%, then │μ2 – μ1│ = 0.3, σ = 0.15 and δ = 2. Hence, m = 20.

The sample size for a power of 80% is estimated to be ≈40 patients. Assuming a power of 90%, the sample size increases to 45 patients.

Cross-sectional validation of the Italian version of the questionnaire was evaluated by calculating the correlation between the FAQLQ–PF and the FAIM–PF. Internal consistency was investigated by calculating Cronbach’s α; we required a correlation score of >70%.

Data collected from the questionnaire were analyzed using a dual approach: as single answers (score from 0 to 6, worsening HRQoL), and grouped as topics (emotional impact; food-related anxiety; social and food limitations). First we considered the results as continuous variables and than we compared binary epidemiological variables using *t*-tests and variance analyses (ANOVA). The five epidemiological variables tested were: sex; age; severity of the child’s allergy (according to the reaction severity grades proposed by Sampson [24]); the number of foods excluded; and the duration of the cow-milk and egg exclusion diets. The chi-square was used to compare proportions with categorical variables. Spearman non-parametric correlation was adopted for ordinal variables or continuous variables subsequently organized into different classes. For each test, p < 0.05 was considered significant.

## Results

Ninety-six children (59 (61.4%) males; median age, 3.92 years (SD + 2.67)) were the study cohort. All participants completed all of the questionnaire. All subjects were already on a therapeutic diet that excluded one or more type of food: milk (n = 81), eggs (n = 57), fish (n = 7), peanuts (n = 1), soybeans (n = 1), wheat (n = 1), rice (n = 1), apples (n = 1) and peaches (n = 1).

Fifty-six children (58.3%) excluded 1 food, 30 children (31.2%) excluded 2 foods, and 10 (10.4%) excluded ≥3 foods (Table 1). All children had undergone skin-prick tests (SPTs) previously. Those younger than 4 years of age used a base panel of allergens: α-lactalbumin, β-lactoglobulin, casein, yolk, egg-white, wheat, soy, *Dermatophagoides pteronyssinus*, *Dermatophagoides farinae*, Alternaria, Cynodon, Lolium, olea, parietaria, cat and dog epithelia. For older children, we added these allergens: peanuts, hazelnuts, corn, apple, peach, kiwi fruit, cocoa, tomato, pine, birch, cypress, and plane.

**Table 1:** Main clinical features of the 96 children with food allergy

		**n. (%)**
Gender	Males	59 (61.4)
Age (years)	<3	43 (44.9)
	<3-6	37 (38.5)
	<6-12	16 (16.6)
Anaphylaxis		24 (25)
Self-injectable epinephrine		8 (8.3)
Symptoms	Skin	82 ( 85.4)
	Airways	46 (47.9)
	Gut	38 (39.5)
Age at onset (months)	<6	66 (68.7)
	≤ 6-12	23 (23.9)
	<12 < 36	7 (7.3)
Food avoided	Milk	81 (84.4)
	Eggs	57 (59.4)
	Fish	7 (7.3)
	Peanuts	1 (1)
	Soybeans	1 (1)
	Wheat	1 (1)
	Rice	1 (1)
	Apples	1 (1)
	Peaches	1 (1)
Number of diets	1	56 (58.3)
	2	30 (31.2)
	3	10 (10.4)

SPTs were positive for food allergens in 96/96 children (100%), and for inhalant allergens in 24/96 children (25%). Eighty-five percent had a history of eczema, 48% suffered from rhino-conjunctivitis or had well-controlled asthma, and 38% suffered from gastrointestinal symptoms. Twenty-four patients (25%) had previously also suffered moderate or severe systemic anaphylactic reactions according to Sampson’s criteria from 2006 [25]. Eight of these (8%) had used a self-injectable epinephrine device. Eighty-eight (92%) of all participants underwent a standardized oral food challenge to confirm their allergy; the 8 children who had severe anaphylaxis and needed epinephrine did not have the challenge.

The onset of symptoms during the first 6 months of life was reported in 66 patients, in 23 patients (23.9%) between 6 months and 12 months, and in 7 children (7.3%) between 12 months and 36 months (Table 1).

### Questionnaire validation

For the FAQLQ–PF, 45 completed questionnaires were used for the cross-sectional validation. A positive correlation between the FAQLQ–PF and FAIM–PF was found (r = 0.47; p < 0.01), thereby supporting the validity of the construct of the FAQLQ–PF. To evaluate internal consistency, we made inter-item correlations by comparing the scores of one or more pair of items that propose to measure the same general construct and which belong to the same area (food anxiety, emotional impact, and social limitations). The internal consistencies calculated (Cronbach’s α) were 0.85, 0.70 and 0.75, respectively, for the individual areas. Good overall internal consistency was found (Cronbach’s α = 0.76).

### QoL parameters

QoL standards were measured and then related to different parameters. We divided the sample into groups of children on the basis of sex, age (0–3, 4–6, 7–12 years), severity of the clinical picture (presence or absence of anaphylaxis episodes), number of foods excluded from the diet (1, 2, 3, >3) and duration of cow-milk and egg exclusion diets (2–24, 25–48, 49–120 months). Mean scores from each area (representing the mean score of all items belonging to the same area) and from the overall HRQoL (which corresponds in turn to the mean of the three previous areas) were compared between the different groups. Sex did not influence the QoL *in toto* or its individual aspects (Table 2).

**Table 2:** Influence of different variables on the total QoL and on single topics

**Variable**	**Total QoL (mean ± D S)**	**Emotional impact (mean ± D S)**	**Food related anxiety (mean ± D S)**	**Social limitations (mean ± D S)**
**Gender**				
**Male**	2.02 (± 1.35)	1.88 (± 1.44)	1.98 (± 1.64)	2.46 (± 1.63)
**Female**	1.80 (± 1.62)	1.77 (± 1.52)	1.69 (± 1.75)	2.20 (± 1.77)
P	ns	ns	ns	ns
Age (y)**				
≤3	1.42 (± 1.36)	1.30 (± 1.37)	1.44 (± 1.72)	2.08 (± 1.59)
>3-6	2.35 (± 1.48)	2.25 (± 1.47)	2.25 (± 1.47)	2.73 (± 1.77)
<6-12	2.37 (± 1.28)	2.34 (± 1.29)	2.53 (± 1.41)	2.25 (± 1.65)
P	F ± 4.3; p < 0.007	F ± 5.80; p < 0.005	F ± 3.0; p < 0.05	ns
Anaphylaxis*				
Yes	2.55 (± 1.39)	2.23 (± 1.37)	2.89 (±1.65)	2.72 (± 1.64)
No	1.73 (± 1.43)	1.71 (±1.49)	1.53 (±1.56)	2.24 (± 1.69)
P	<0.02 t ± 2.5; p < 0.02	ns	<0.001 t ± 3.6; p <0.001	ns
n. of diets**				
1	1.82 (± 1.46)	1.80 (± 1.43)	1.62 (± 1.52)	2.25 (± 1.80)
2	1.94 (± 1.51)	1.72 (± 1.58)	2.17 (± 1.93)	2.40 (± 1.48)
3	2.54 (± 1.20)	2.42 (± 1.29)	2.37 (± 1.65)	2.82 (± 1.60)
P	ns	ns	ns	ns
2-24	1.24 (± 1.09)	1.11 (± 1.00)	1.11 (± 1.34)	1.97 (± 1.49)
25-48	2.03 (± 1.43)	1.88 (± 1.42)	2.05 (± 1.78)	2.42 (± 1.59)
49-120	2.67 (± 1.46)	2.66 (± 1.53)	2.50 (± 1.53)	2.86 (± 1.91)
P	<0.001 F ± 6.1; p < 0.001	<0.001 F ± 6.5; p < 0.001	<0.0006 F ± 7.2; p < 0.0006	ns
Egg free diet** (length, months)				
2-24	1.73 (± 1.71)	1.55 (± 1.79)	1.86 (± 2.09)	2.20 (± 1.70)
25-48	2.20 (± 1.57)	1.80 (± 1.52)	2.25 (± 1.94)	2.70 (± 1.80)
49-120	2.07 (± 1.00)	2.17 (± 1.08)	2.00 (± 1.03)	2.60 (± 1.54)
P	ns	ns	ns	ns

*Using *t*-test.

**Using one-way ANOVA.

### Age-related QoL

Children aged 3–6 years and those aged 7–12 years had worse scores (Likert scale) than patients younger than 3 years for the total HRQoL (p < 0.007), food-related anxiety (p < 0.05) and emotional impact (p < 0.005). The area of social constraints showed consistently high scores but without significant differences between different age groups (Table 2).

### Severity of reactions and QoL

Previous episodes of anaphylaxis had a higher impact on the HRQoL *in toto*. The score was worse for the 24 children who had experienced anaphylactic reactions compared with the 72 children who did not (p < 0.02). Individual analyses of the three aspects that constitute the HRQoL were evaluated in detail. Food-related anxiety in the group of children who had reported anaphylaxis showed significantly higher scores compared with the others (p < 0.001). Emotional impact appeared to be influenced slightly more within the first group, but not in a significant manner. Finally, there were high scores for social and diet constraints but no significant differences between groups were reported (Table 2).

### Duration of exclusion diet and QoL

The duration of the exclusion diet had a negative effect on the HRQoL, but significant differences were observed only among patients on the milk-exclusion diet. Considering the 81 children on a cow milk-free diet, there was a direct correlation between the duration of the diet and the HRQoL score. When the duration increased, the Likert’s scale score also increased (p < 0.001), mainly with regard to food-related anxiety (p < 0.006) and emotional impact (p < 0.001). The scores for social constraints did not show significant differences between groups because they were high regardless of the duration of the exclusion diets. The egg-free diet did not show meaningful impairment upon HRQoL standards (Table 2). We assessed the HRQoL only in relation to the duration of milk- and egg-exclusion diets because of the low number of exclusion diets for other foods in our study cohort.

## Discussion

The exclusion diet is the main treatment for food-related allergies. Whether to eliminate the offending food is controversial because alternative approaches are gaining interest [26-28]. We assessed Italian children with food allergies who were on one or more exclusion diet. This was the first study in an Italian population that evaluated the impact and consequences of exclusion diets on the HRQoL of patients and their families using a questionnaire that compared three age groups. The questionnaire showed good internal consistency.

Cummings et al. [29] undertook a review of studies published in English from 1990 to 2009 on the impact of food allergy on affected children, adolescents and their families. They observed that the constant vigilance needed to avoid allergens and the daily management of food allergy impacted on daily family activities and social events. In accordance with those findings, all groups surveyed in our study showed high scores with regard to social limitations. This finding suggested that avoidance of places related to food and ongoing environmental monitoring are preventive measures taken by all families of children with food allergies.

In our experience and as shown previously [30,31], the independent variable “experience of severe anaphylaxis” is associated with significantly higher scores in “anxiety related to food”, which is the dependent variable with the heaviest impact upon an impaired HRQoL. Children who have had anaphylaxis reactions are very fearful of life-threatening food reactions. These severe episodes cause acute illness followed by severe psychological trauma, which is renewed constantly by the fear of being re-exposed unexpectedly to the offending food. In addition, the uncontrolled feeling of anxiety and fear transferred by parents to the child can cause these patients to treat each meal as a “recurrent danger”, thereby eliciting a bad relationship with food.

Munoz et al. [32] reported that children who have experienced a severe allergic reaction may became withdrawn and fearful, or develop disordered eating patterns. These findings are not in accordance with other studies [14-33]. Cummings et al. [33] assessed 41 children with nut allergies. Some children had an IgE-mediated allergy to the nuts and reacted to this food, others had not suffered previous reactions to the nuts and were enrolled on the basis of highly predictive SPTs or serum levels of IgE. Their results showed that the severity of previous reactions, the need for treatment with epinephrine, and previous hospital admissions due to a reaction had no significant effect on maternal- or child-reported QoL or anxiety. Marklund et al. [14] studied 134 children with parent-reported food hypersensitivity and observed better psychosocial wellbeing for children reported to react to food with difficulty in breathing and/or anaphylaxis.

Age appears to be another variable that affects the HRQoL. Children under 3 years of age are guided entirely by their parents with regard to nutrition, are less affected by social limitations, and are virtually unaware of food restrictions. Increased awareness and independence acquired in childhood and adolescence induce a higher score on the Likert scale, and therefore have a real emotional impact, anxiety and then a worse perception of the HRQoL. Wassemberg et al. [31], using the FAQLQ–PF, reported a worse QoL in school-aged children compared with younger children (0–3- and 4–6-years-old) and provided a picture of HRQoL changes upon aging.

Studies have shown that having more food allergies increases the impact on the HRQoL [15,34]. In the present study, the number of allergens excluded was not as a relevant factor to modify the HRQoL. In fact, although there was a trend of higher scores in all HRQoL domains, statistically significant results were not obtained. This observation suggests that children are more concerned with the diet itself rather than the extent of limitations. It also highlights that the fear of accidentally ingesting an unrecognized food allergen is the main cause of anxiety and induces the perception of being “sick”.

The most interesting and original data regard the duration of cow milk- and egg-free diets. Avoiding milk (but not eggs) affects the HRQoL differently depending on the duration of food restriction. The longer the duration of the milk-free diet, the higher the score. Consequently, the emotional experience and food-related anxiety in the HRQoL worsen dramatically. Conversely, the duration of an egg-free diet does not affect the HRQoL. A possible explanation is that dairy products are a major part of the Italian diet, more so than eggs. Hence, their exclusion for long periods can lead to many restrictions, and this increases stress in children and in their families.

Our study has limitations. First, the study cohort was a small number of participants selected from a single outpatient setting. Hence, we could not generalizations about our findings. Second, the validity of the construct of FAQLQ–PF was demonstrated only by comparing FAQLQ–PF with FAIM–PF. The use of other scales would be useful to confirm the validation of the Italian questionnaire. In the future, it would be interesting to evaluate the HRQoL in Italian schoolchildren and adolescents with food allergy using specific questionnaires completed by the schoolchildren/adolescents themselves.

## Conclusions

Exclusion diets are not free of side effects, and may significantly impair the HRQoL of Italian children in the same way as has been revealed in studies of other populations. Exclusion diets should be administered only for the period of time that is needed. Certain subgroups of patients (school-aged children, those with severe reactions, or those on extended milk-exclusion diets) experience a greater impact on their QoL. Maintaining high levels of the HRQoL, especially in these subgroups, may facilitate clinical pathways for healing and acceptance of the disease. It may also facilitate early detection in children with special needs, enabling help and support to be provided in a timely fashion. The validation in Italian of the questionnaire used here will provide the opportunity to assess the HRQoL of other Italian children with this disease.

All the data reported so far illustrate the importance of taking into account different approaches when managing food allergy. Oral immunotherapy appears to be a promising option for dealing with food allergy. Avoiding extended diets and reducing the risk of anaphylaxis from hidden allergens is possible, but further studies are required to assess the benefit and safety of these alternative therapies [35,36].

## Competing interests

We declare that there are no known conflicts of interest associated with this publication and there has been no significant financial support for this work that could have influenced its outcome.

## Authors’ contributions

LI and MD developed the design of the study and selected patients with food allergy to be enrolled. LB provided psychological support to families while they completed questionnaires and drafted the manuscript. AMZ and GDC developed the design of the study and drafted the manuscript. GT participated in the design of the study and conducted statistical analyses. VDV administered the QoL questionnaire and helped with statistical analyses. All authors approved the final version of the manuscript.

## Pre-publication history

The pre-publication history for this paper can be accessed here:

http://www.biomedcentral.com/1471-2431/13/203/prepub
